# The breadth of primary care: a systematic literature review of its core dimensions

**DOI:** 10.1186/1472-6963-10-65

**Published:** 2010-03-13

**Authors:** Dionne S Kringos, Wienke GW Boerma, Allen Hutchinson, Jouke van der Zee, Peter P Groenewegen

**Affiliations:** 1 NIVEL-Netherlands Institute for Health Services Research, Otterstraat 114-118, 3500 BN Utrecht, the Netherlands; 2 ScHARR-School of Health and Related Research, University of Sheffield, Regent Court, 30 Regent Street, Sheffield, S1 4DA, UK; 3 Department of Human Geography, Department of Sociology, University of Utrecht, PO Box 80140, 3508 TC Utrecht, The Netherlands; 4 Faculty of Health Sciences, Department of International Public Health, University of Maastricht, PO Box 616, 6200 MD Maastricht, The Netherlands

## Abstract

**Background:**

Even though there is general agreement that primary care is the linchpin of effective health care delivery, to date no efforts have been made to systematically review the scientific evidence supporting this supposition. The aim of this study was to examine the breadth of primary care by identifying its core dimensions and to assess the evidence for their interrelations and their relevance to outcomes at (primary) health system level.

**Methods:**

A systematic review of the primary care literature was carried out, restricted to English language journals reporting original research or systematic reviews. Studies published between 2003 and July 2008 were searched in MEDLINE, Embase, Cochrane Library, CINAHL, King's Fund Database, IDEAS Database, and EconLit.

**Results:**

Eighty-five studies were identified. This review was able to provide insight in the complexity of primary care as a multidimensional system, by identifying ten core dimensions that constitute a primary care system. The structure of a primary care system consists of three dimensions: 1. governance; 2. economic conditions; and 3. workforce development. The primary care process is determined by four dimensions: 4. access; 5. continuity of care; 6. coordination of care; and 7. comprehensiveness of care. The outcome of a primary care system includes three dimensions: 8. quality of care; 9. efficiency care; and 10. equity in health. There is a considerable evidence base showing that primary care contributes through its dimensions to overall health system performance and health.

**Conclusions:**

A primary care system can be defined and approached as a multidimensional system contributing to overall health system performance and health.

## Background

The WHO World Health Report 2008, entitled 'Primary health care now more than ever', has clearly articulated the need to mobilize the production of knowledge on primary care [1]. Even though there is general agreement that primary care is the linchpin of effective health care delivery [2-5], to date no efforts have been made to systematically review the scientific evidence underlying this supposition.

The investment in primary care reforms by governments and international agencies such as the World Bank and the WHO has been substantial. In particular in countries with health care systems in transition, joint investment programmes between governments and non governmental organisations have been established [6-8]. Also from the wealth of charters, resolutions, and statements that continue to originate from governments and non-governmental organizations worldwide, it is evident that policymakers are concerned about improving the development of primary care systems [1,9]. The most recent example is Resolution WHA62.12 which was accepted in May 2009 at the 62^nd ^World Health Assembly, which urges WHO member states to strengthen their health care systems through the values and principles of primary care.

Despite such significant reliance and investment in boosting primary care development, there is a lack of detail in documents regarding what constitutes an effective primary care system, and what its evidence base is. The available evidence for the importance of primary care is limited to the work of Barbara Starfield. Starfield's instrument examines essential 'components' of primary care on a general, aggregate (macro) level. Each component is measured by one indicator, using a scoring system ranging from 0 to 2. However, when the objective of a study is to capture the complexity behind the primary care components, more detailed, process-oriented, and explanatory indicators are needed for each component.

Moreover, so far little attention has been paid to systematically monitoring primary care development in Europe. This hinders identification and sharing of experiences and keeps the lessons learned scarce [1,6-10].

Creating an effective primary care system is not a question of implementing one recipe since systems are context dependent. Their development is to a large part shaped by a country's historical background, welfare state, health problems, characteristics of the health care system, and societal values and beliefs. Therefore, the strength of a country's primary care system is determined by the degree of development of a combination of core primary care dimensions in the context of its health care system [11,12].

This study aims to examine the breadth of primary care systems in Europe by identifying their core dimensions and to assess the evidence for their interrelations and their relevance to outcomes at (primary) health system level.

## Methods

### Search strategy

The following electronic databases were searched between April and July 2008: MEDLINE, Embase, Cochrane Library, CINAHL, King's Fund Database, IDEAS Database, and EconLit. For practical reasons such as time and financial constraints, the search was limited to publications published between January 2003 and July 2008, written in English, and including an abstract. Clinical trials and editorials were excluded.

The search consisted of two stages. Stage 1 was restricted to reviews on the following topics: access, continuity, coordination, comprehensiveness, and context orientation. The topics were based on the frequently used definition by Starfield et al. [13] defining primary care as the provision of integrated, accessible health care services by clinicians who are accountable for addressing a large majority of personal health care needs, developing a sustained partnership with patients, and practicing in the context of family and community. This search strategy was an efficient method to cover the extensive primary care literature area. An additional advantage of this method was that it let to an overview of key primary care study results that went beyond the 5 year time restriction. Stage 2 was an open search (due to a lack of reviews) on (primary) health system performance measurement and accountability. The search strategy included a combination of text words and Medical Subject Headings (MeSH) terms relating to these topics of interest, searched in titles and abstracts of studies. To focus the search, studies were only included if their 'Major Topic Headings' included a primary care keyword or one of the sub-topics of interest (access, etc.). The search strategy was devised for use in MEDLINE (accessed via PubMed) and adapted for other databases (see Additional file 1).

### Methods of screening and selection criteria

The applied review strategy was guided by a manual for performing systematic literature reviews on a health services research topic [14].

An initial screening of studies was based on titles, performed by one researcher. In the second screening, titles and abstracts were evaluated by two reviewers independently. Finally, the full texts of the studies were assessed for inclusion, also by two reviewers independently. Any discrepancies between reviewers were resolved through discussion.

We aimed to identify studies describing, measuring, or explaining the (health or health system performance) impact of dimensions of primary care systems in Europe. We therefore excluded studies that focussed on: (a) low income countries (gross national income per capita 975 USD or less); (b) personal opinions; (c) small scale studies; (d) other topics than primary care system dimensions (functions, services, professionals, indicators); (e) (primary) health care functions without mentioning of implications for primary care structures, organization or performance. The final list of included studies was evaluated for their completeness by a panel of 10 primary care experts from 9 European countries (mostly senior researchers and general practitioners) who participate in the EC funded project Primary Health Care Activity Monitor for Europe (PHAMEU, see http://www.phameu.eu). This evaluation led to two additions to the publication list [15,16].

### Data extraction

The following information was abstracted from the studies that met our study criteria: setting, sample size, study design, study focus, primary care dimensions studied, identified associations between primary care dimensions and health system performance or health. The articles were grouped by the primary care dimension(s) they addressed.

The quality of the original articles was assessed by two reviewers. The articles were scored on their internal validity ranging from 1 (very strong internal validity established by approaches, very strong statistical power, solid explicit analysis of the introduction and context) to 4 (weaker internal validity supported by primarily non-experimental approach with or without explicit reference to intervention and context). The external validity of the articles were scored ranging from 1 (very strong external validity supported by a large study population, random sample, and explicit analysis of context and intervention factors for which generalization is possible) to 4 (weaker external validity based on weak or selective reference population, and weak intervention and context reference).

Given the strong reliance in this study on literature reviews, a clear distinction was made between evidence resulting from single studies and from literature reviews. The results section on evidence for the interrelations of dimensions and associations with outcomes, only reported evidence from literature reviews.

## Results

### Study characteristics

**Figure 1:**
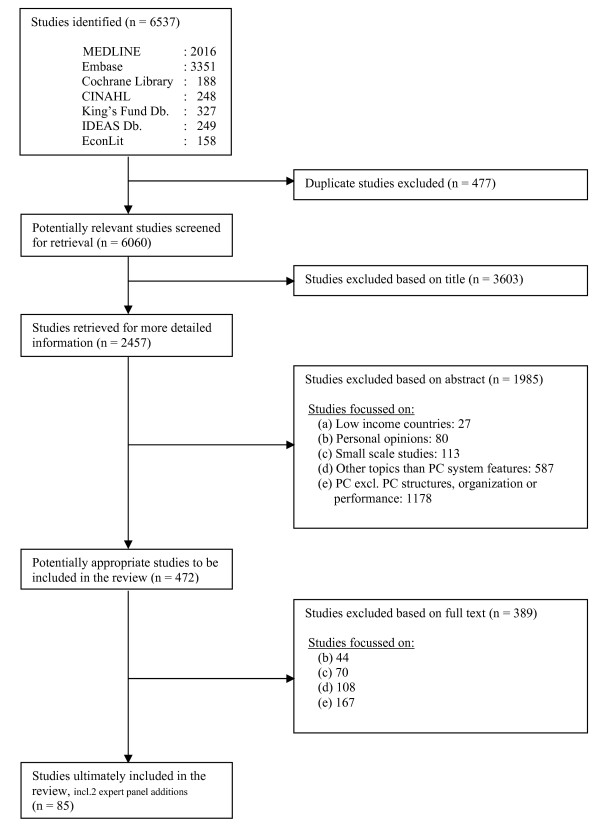
**Study selection process**.

A total of 6537 publications were identified; of these 477 were duplicates. 2457 were selected for further scrutiny on the basis of screening the titles. Following a review of the abstracts, the full text of 472 publications were retrieved, and assessed on their fulfilment of the selection criteria. Among the end references of the remaining 83 studies, two additional studies were identified by the international panel of primary care experts that met the study criteria. 85 publications were finally included in the current evaluation (figure 1).

Additional file 2 provides a descriptive overview of the included studies. Thirty-five were cross-sectional studies [4,17-50] with on average a fairly strong internal validity (score 3.5) and a strong external validity (score 2.5). Twenty-five were literature reviews [13,51-74]. Thirteen were descriptive studies [16,75-84] with on average a weaker validity (score 4). Five were prospective cohort studies [85-89], four were retrospective cohort studies [15,90-92] with a fairly strong internal and external validity (score of 3.5 and 2.8 respectively). Three were cost-benefit studies [93-96] with a weaker validity (score 4)

Primary care was the subject of studies in a wide range of countries. There were forty-five single country studies [15-17,19-22,24-31,34,35,37,38,40-46,57,59,75,77,82-93,95-97]. Of these, twelve were situated in the United Kingdom, nine in the United States, four in Australia, four in Canada, three in Spain, two in the Netherlands, two in Norway, and the rest in Belgium, Croatia, Cyprus, Finland, Greece, New Zealand, Poland, Serbia and Switzerland. Sixteen international comparative primary care studies were included, covering forty-eight countries [4,18,23,32,33,36,39,47-50,63,76,78,79,81]. The remaining twenty-four studies had an unrestricted setting [13,51-56,58,60-62,64-74,80,94].

### The core dimensions of primary care

**Figure 2:**
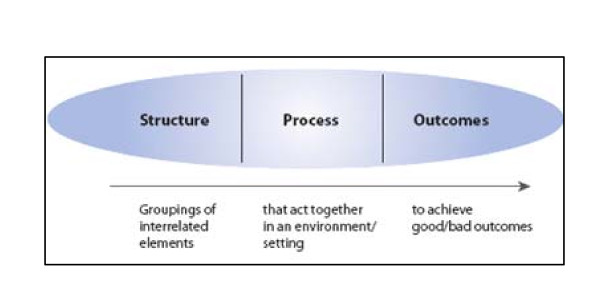
**Framework of structure, process, outcomes**.

Primary care can be approached as a system consisting of three complex levels (structure, process and outcome) which each consist of several dimensions (figure 2) [98]. Previous studies have shown the suitability of this approach for primary care systems [e.g. [83,99,100]].

To identify the dimensions, each study was grouped according to the similarities in primary care features they studied on one or more levels of the primary care system. Each group of studies was then labelled with an appropriate dimension (see Additional file 2). A primary care dimension is a major subject area consisting of several primary care system features. Primary care system features are the key attributes of a primary care system dimension. A dimension (at a higher level) can consist of one or more features, depending on its complexity. It was taken into account that publications could focus on multiple primary care dimensions. Table 1 provides an overview of studies per dimension.

**Table 1:** Identified dimensions of PC systems

Dimensions of PC systems	Studied by
***Level: PC Structure***	
Governance of the PC system	[4,13,15,16,21,23,28,36,38,43,46,51,59,63,64,68,74,77,79,82-85,87,96,97]
Economic conditions of the PC system	[4,13,16,18,30,34,38,44,47,54,75,84,90,94]
PC Workforce development	[4,13,16,21,23,31,36,38,46,48,49,51,55,59,72,76,80-83,90]
***Level: PC Process***	
Access to PC services	[4,13,16,19,20,23,25,28,29,38,43,45,46,49,53,54,57,61,65,68,72,75,78,80,82,89,91,95]
Continuity of PC	[4,13,17,19,22,23,27-29,31,35,37,40,42,43,45,48,51,56,60,65-67,69-71,73,80,84,86,88]
Coordination of PC	[4,13,17,18,20,24-26,28,31-33,41-43,45,46,48,50,55,58,65,67,69,71,74,82,84,92-94]
Comprehensiveness of PC	[4,13,23,28,31,45,50,51,62,65,68,71,80,83,84]
***Level: PC outcome***	
Quality of PC	[4,13,16,20,23-26,28,29,32,39,51-54,62,68,72,75,80,82,91]
Efficiency of PC	[18,28,29,38,43,47,54,57,68,72,75,82,91,94]
Equity in health	[28,68,77]

The *structure *of a primary care system consists of three dimensions: 1) Governance; 2) Economic conditions; 3) Workforce development. The primary care *process *is determined by four dimensions: 4) Access; 5) Continuity of care; 6) Coordination of care; 7) Comprehensiveness of care. The *outcome *of a primary care system includes three dimensions: 8) Quality care; 9) Efficiency of care; 10) Equity in health.

The applied definitions of each of the dimensions and available evidence of their interrelations and association with (primary) health care system outcomes will be discussed separately by dimension in the next sections.

### Governance of the primary care system

The governance dimension can be summarised as the vision and direction of health policy exerting influence through regulation, advocacy, collecting and using information. Eight features of primary care governance were identified:

*1. Health (care) goals: *The vision and direction of a primary care system depend on explicit health or health care goals at national level [68,83].

*2. Policy on equity in access to primary care services: *Equity in access can be influenced by policy development and regulation on the distribution of human resources and quality of care across geographical areas, by setting policy objectives regarding the duration of waiting time for (specific) primary care services; and by assuring universal financial coverage for primary care services by a publicly accountable body [4,13,28,46,68,82,83].

*3. (De)centralization of primary care management and service development: *This is shaped by the level (national, regional, local) at which primary care policies are determined, the degree in which standards allow for variation in primary care practices geographically, and the development of policies on community participation in primary care management and priority setting [4,28,45,59,77,82,96].

*4. Quality management infrastructure *in primary care: This can consist of a number of mechanisms that need to be in place to assure adequate quality of care. These include coordination of quality management, quality assessment mechanisms, certification of providers, licensing of facilities, quality incentives, availability of quality information, availability of relevant clinical guidelines, professional competence and standardization of facility equipment [15,16,23,28,36,38,43,49,51,59,63,64,79,83-85,87,96].

*5. Appropriate technology in primary care: *Medical technology in terms of techniques, drugs, equipment and procedures are crucial in the delivery of primary care. Appropriate development and use can be stimulated at governmental level by developing a national policy or strategy concerning the application of ICT in primary care, and by organizing guidance to government and providers on technology appraisal on the use of new and existing medicines and treatments [16,35,77].

*6. Patient advocacy: *This can be embedded by primary care-oriented patient organisations, and patient compliance procedures in care facilities [28,46,83].

*7. Ownership status of primary care practices: *This provides an indication of the level of government involvement in primary care provision [21,97].

*8. Integration of primary care in the health care system: *Integration of primary care through interdisciplinary collaboration between primary care and secondary care, and task substitution and delegation can be promoted by governmental integration programmes, or legislation [28,59,74].

#### Evidence for the relevance of the primary care governance dimension

Additional file 3 provides an overview of the key findings for primary care governance and its relation with (other) primary care dimensions and (primary) health care system outcomes. Studies found associations with access, continuity, coordination, comprehensiveness, quality, equity in health, efficiency, population health, local accountability, quality of professional life, patient satisfaction, costs, and the strength of primary care systems. The evidence was based on ten single (original research) studies and one literature review.

The literature review by Starfield et al. [13] found that primary care-supportive governmental policies improve access of care, continuity and coordination of care, and the delivery of wide range of services, in particular preventive care, and achieving equity in health. Consistent governance features of strong primary care systems were pro-equity policies; universal financial coverage; and limiting patient cost sharing for primary care services.

### Economic conditions of the primary care system

The economic condition of a primary care system is made up of six features:

*1. Health care funding system*: The method of financing health care for the majority of the population, such as taxes, health insurance, or private means [4,13].

*2. Health care expenditures: *Total expenditures on health care [16,75,84].

*3. Primary care expenditures: *Total expenditures on primary care [16,75,84].

*4. Employment status of primary care workforce: *Such as salaried employed providers, or self-employed providers with/without contract(s) with health service or insurance [90].

*5. Remuneration system of primary care workforce: *Such as fee-for-service payment, capitation payment, salary payment or mixed payment [34,44,47,94].

*6. Income of primary care workforce*: Annual income of primary care workforce, also compared to specialists [13,16,38].

#### Evidence for the relevance of the economic conditions of a primary care system

Additional file 4 provides an overview of the key findings for the economic conditions of a primary care system and its relation with (other) primary care dimensions and (primary) health care system outcomes. Studies found associations with access, continuity, comprehensiveness, quality, efficiency, population health, and quality of professional life. The evidence was based on seven single studies.

### Primary care workforce development

The workforce development dimension can be summarised as the profile of primary care professionals that make up the primary care workforce, and the position that they take in the health care system. The following six features of this dimension were identified:

*1. Profile of primary care workforce: *The type of health care professionals that are considered to be part of the primary care workforce, and their gender balance [4,13,31,51,55,72,76,80,82].(

*2. Recognition and responsibilities: *Whether the primary care discipline is officially recognized as a separate discipline among the medical disciplines, with recognised responsibilities [23,49,76].

*3. Education and retention: *Vocational training requirements for primary care professionals, primary care workforce supply and retention problems, and capacity planning [4,13,36,49,81,83,90].

*4. Professional associations: *The organization of professional associations for the primary care workforce [59].

*5. Academic status of the primary care discipline: *Reflected by academic departments of family medicine/primary care within universities [49].

*6. Future development of the primary care workforce: *Hampering threats to the current development and expected trends in the future development of the primary care workforce, from the point of view of stakeholders [49].

#### Evidence for the relevance of primary care workforce development

Additional file 5 provides an overview of the key findings for the development of the primary care workforce and its relation with (other) primary care dimensions and (primary) health care system outcomes. Studies found associations with access, continuity, comprehensiveness, and efficiency of primary care. The evidence was based on three single studies [38,48,82] and two literature reviews [59,72].

The literature review by Wilson and Childs [72] showed that the gender balance of the primary care workforce can influence access, continuity and efficiency of care, and the scope of services delivered. Halcomb et al. [59] found that the availability of practice nurses in general practice increases the comprehensiveness of services provided.

### Access to primary care services

Access to primary care services can be defined in terms of seven features:

*1. Availability of primary care services: *The volume and type of primary care services relative to population needs [13,16,28,38,49,57,91].

*2. Geographic accessibility of primary care services: *Remoteness of services in terms of travel distance for patients [20,91].

*3. Accommodation of accessibility: *The manner in which resources are organized to accommodate access (e.g. appointment system, after-hours care arrangements, home visits) [13,19,23,28,29,45,46,57,61,72,75,78,89,91,95].

*4. Affordability of primary care services: *Financial barriers patients experience to receive primary care services, such as co-payments and cost-sharing arrangements [4,13,68,91].

*5. Acceptability of primary care services: *Patient satisfaction with the organization of primary care [25,43,91].

*6. Utilisation of primary care services: *Actual consumption of primary care services [43,57].

*7. Equality in access: *The extent to which access to primary care services is provided on the basis of health needs, without systematic differences on the basis of individual or social characteristics [28,46,54,57].

#### Evidence for the relevance of access to primary care services

Additional file 6 provides an overview of the key findings for access to primary care services and its relation with (other) primary care dimensions and (primary) health care system outcomes. Studies found associations with continuity, comprehensiveness, quality, equity in health, population health, quality of professional life, patient satisfaction, costs and strength of primary care. The evidence was based on six single studies and six literature reviews.

Wilson and Childs' literature review [72] showed that the consultation length influences the continuity of care by the quality of medical recordkeeping, and patient enablement. Two reviews [13,72] found that physician supply and consultation length influence the range of services provided in primary care. The influence of access on the provided quality of care (lower hospitalization rates for ambulatory care sensitive conditions (ACSCs), prescribing quality) was confirmed by four reviews [13,53,61,72]. It was also consistently shown that access can reduce socio-economic and racial disparities in health [13,57]. Three reviews found positive associations between accessibility of care and population health [13,53,65]. Physician workload and stress are influenced by access arrangements and consultation length [61,72]. Two reviews showed associations between patient satisfaction, and consultation length and access arrangements [61,65]. It was also shown that a greater supply of family physicians is associated with lower total costs of health services [13]. Starfield et al. [13] concluded that access was a core dimension of a strong primary care system

### Continuity of primary care

The continuity of care dimension can be summarised as a hierarchy of three features:

*1. Longitudinal continuity of care: *Having a long-term relationship between primary care providers and their patients in their practice beyond specific episodes of illness or disease [4,13,17,19,22,27,37,40,42,45,48,56,60,66,70,71,73,84,86].(Some definitions also speak of personal or family continuity, where the continuity of care between a single provider or a family is stressed [4,13,28,45,48,66,70].

*2. Informational continuity of care: *An organized collection of each patient's medical information readily available to any health care provider caring for the patient. This can be reached through medical record keeping, clinical support and referral systems [23,28,31,35,37,45,48,51,66,67,69-71,73,88].

*3. Relational continuity of care: *The quality of the longitudinal relationship between primary care providers and patients, in terms of accommodation of patient's needs and preferences, such as communication and respect for patients [13,28,29,37,43,45,48,66,70,73].

The existence of a consistent and coherent approach to the management of a health problem, also known as 'management continuity', is sometimes added to this list of features [28,48,70,73]. However, this shows overlap with the coordination of care dimension.

#### Evidence for the relevance of continuity of primary care

Additional file 7 provides an overview of the key findings for continuity of primary care and its relation with (other) primary care dimensions and (primary) health care system outcomes. Studies found associations with coordination, comprehensiveness, quality, efficiency, population health, patient satisfaction, costs, and strength of primary care. The evidence was based on six single studies and seven literature reviews.

The literature review by Cabana and Jee [56] found a positive association between continuity of care and improved care coordination. Continuity of care was consistently related to improved receipt of preventive services, as shown by four reviews [13,56,60,73]. There was also strong evidence for the relevance of continuity of care to assure receipt of high quality of care, for example in terms of decreased hospitalizations and improved early diagnoses [13,56,60,70,73]. Three reviews agreed that continuity of care is cost-effective in primary care, and ensures greater efficiency of services [13,65,73]. There was also a strong evidence-base for the relation between continuity of care and improved patient satisfaction [13,56,60]. Starfield et al. [13] found that continuity of care is a core dimension of a strong primary care system.

### Coordination of primary care

The coordination of care dimension reflects the ability of primary care providers to coordinate use of other levels of health care [4]. The following features were identified from coordination of care studies:

*1. Gatekeeping system: *The level of direct access for patients to health care providers without a referral from a primary care provider [4,13,33,43,46,94].

*2. Primary care practice and team structure: *Such as shared practices, team premises and team size and tenure [20,24,31,42,74].

*3. Skill-mix of primary care providers: *Diversification and substitution of primary care providers [20,42,55,69,71,74,82,92,93].

*4. Integration of primary care-secondary care: *Careintegration can be achieved through specialist outreach models and clinical protocols facilitating shared care [25,45,46,58,67].

*5. Integration of primary care and public health: *The extent to which primary care providers collaborate with practitioners from the public health sector to provide services that influence health [28,32].

#### Evidence for the relevance of coordination of primary care

Additional file 8 provides an overview of the key findings for coordination of primary care and its relation with (other) primary care dimensions and (primary) health care system outcomes. Studies found associations with access, continuity, comprehensiveness, quality, efficiency, population health, patient satisfaction, costs, and primary care strength. The evidence was based on 14 single studies and ten literature reviews.

The literature review by Chapman et al. [57] found that coordination of care through the application of skill mix can affect different features of access. Five reviews [55,67,69,71,74] found a positive association between coordination and continuity of care. Starfield et al. [13] showed that coordination of care is related to the comprehensives of primary care services, particularly preventive care and health promotion. Studies consistently found a relation between coordination of care and higher quality of care [13,58,59,67,74]., and increased efficiency of care [58,69,74]. Coordination of care had mixed results with respect to health [58,65]. Stille et al. [69] found that both physicians and patient satisfaction were associated with certain features of coordination of care. Coordination of care was also associated with reduced patient costs [67]. Starfield et al. [13] found that coordination of care is positively associated with primary care strength.

### Comprehensiveness of primary care services

Comprehensiveness of primary care services represents the range of services available in primary care to meet patients' health care needs [4,13,28,45,83]. A distinction can be made between:

*1. Medical equipment available: *Range of medical equipment available in primary care practices [23,51].

*2. First contact for common health problems: *Range of health problems for which first contact care in primary care is provided [13,45,84].

*3. Treatment and follow-up of diagnoses: *Range of diagnoses for which treatment and follow-up care is provided in primary care [13,45,50,62,71,80,84].

*4. Medical technical procedures and preventive care: *Range of medical technical procedures and preventive care provided in primary care [13,45,62,71,84].

*5. Mother and child and reproductive health care: *Range of mother and child and reproductive health care services provided in primary care [45,62,71,80,84].

*6. Health promotion: *Range of health promotion activities provided in primary care [13,31,45,62,71,80,84].

#### Evidence for the relevance of primary care comprehensiveness

Additional file 9 provides an overview of the key findings for primary care comprehensiveness and its relation with (other) primary care dimensions and (primary) health care system outcomes. Studies found associations with quality, efficiency, equity, population health, and primary care strength. The evidence was based on one single study [80] and four literature reviews [13,65,68,71].

The literature study by Starfield et al. [13] consistently found that lower hospitalization rates for ACSCs are associated with a comprehensive scope of primary care services. Two reviews [13,65]. found that preventive health care activities are cost-effective in the primary care setting. Early detection and prevention of progression of illness was shown to be related to reduced disparities in severity of illness [68]. The delivery of a wide range of services by primary care providers was related to improved health [13,65,71]. Comprehensiveness of care was shown to be positively associated with primary care strength [13].

### Quality of primary care

The quality of primary care resembles the degree to which health services meet the needs of patients, and standards of care [16,28,32].

This dimension mirrors the quality of the services provided in primary care:

*1. Prescribing behaviour of primary care providers: *Such as the frequency at which providers prescribe medicine [25,51,72].

*2. Quality of diagnosis and treatment in primary care: *For example reflected by the occurrence of avoidable hospitalization for acute ACSCs [52,62,68,91].

*3. Quality of management of chronic diseases: *For example the prevalence of chronic diseases, receipt of treatment characteristics, and the occurrence of avoidable hospitalization for chronic ACSCs [24-26,39,52,62,68,80].

*4. Quality of mental health care: *Such as prevalence of mental disorders, and anti-depressant medication, and continuity of mental care [13,24-26,50].

*5. Quality of maternal and child health care: *Reflected for example by maternal mortality rates, occurrence of preventive screening for pregnant women, and infant vaccination [4,13,62,68].

*6. Quality of health promotion: *Such as obesity, smoking or alcohol use in the population [62,68].

*7. Quality of preventive care: *Such as the occurrence of preventable ACSCs, or cancer screening [24,26,52,62,68,75].

Some studies also include responsiveness or patient-centeredness as a feature of quality of care, which is more subjective and dependent on patients' preferences and expectations [28,32,54,82].

#### Evidence for the relevance of quality of primary care

Additional file 10 provides an overview of the key findings for quality of primary care and its relation with (other) primary care dimensions and (primary) health care system outcomes. Studies found associations with governance, access, continuity, coordination, efficiency, population health, and primary care strength. The evidence was based on two single studies and four literature reviews.

Ansari [52,53] found that reduced quality of primary care, in terms of preventable hospitalizations and ACSCs are an indication for potential inadequacies in primary care, which can be related to mal distribution of primary care resources, barriers to access, problems in continuity of care, and inefficient use of resources. There is insufficient evidence to link prescribing volume to quality of primary care, without evidence of appropriateness [72]. Starfield et al. [13] found a positive association between quality of primary care and health, particularly for indicators in early childhood. Quality of primary care was consistently shown to be associated with primary care strength [13].

### Efficiency of primary care

Efficiency of primary care is the balance between the level of resources in the system used to treat patients to come to certain outcomes [18,54]. Primary care studies approach efficiency in different ways:

*1. Allocative and productive efficiency: *Respectively, minimizing patient's opportunity cost of time spent in treatment; maximizing the patient's outcome, minimizing the cost per patient [28,94].

*2. Technical efficiency: *A system is technical efficient if it cannot reduce its resource use without reducing its ability to treat patients or to reach certain outcomes [18].

*3. Efficiency in performance of primary care workforce: *Reflected by basic figures relating to the provision of care, such as number of consultations and their duration, frequency of prescription medicines (unnecessary use), and the number of new referrals to medical specialists [38,43,47,57,72,91].

#### Evidence for the relevance of efficiency of primary care

Additional file 11 provides an overview of the key findings for primary care efficiency and its relation with (other) primary care dimensions and (primary) health care system outcomes. Studies found associations with economics, workforce development, access, continuity, coordination, comprehensiveness, and quality. The evidence was based on five single studies, and seven literature reviews.

The literature review by Wilson and Childs [72] found that female GPs investigate more and prescribe less than male GPs. Two reviews [13,65]. agreed that continuity of care in primary care ensures greater efficiency of services. Coordination of care, in terms of team size and composition, and specialist outreach in primary care are associated with cost-effective care, and better health [58,69,74]. The reviews by Sans-Corrales et al. [65] and Starfield et al. [13] found that preventive health care activities are cost-effective in the primary care settings. Inefficient use of resources in primary care is associated with preventable hospitalizations and ACSCs [52].

### Equity in health

Equity in health seems to be a relatively small, though important area of research in primary care. It is the absence of systematic and potentially remediable differences in health status across population groups [28,68]. It is approached by the level of disparity for primary care sensitive health outcomes across population groups [68,77].

#### Evidence for the relevance of equity in health

Additional file 12 provides an overview of the key findings for equity in health and its relation with (other) primary care dimensions and (primary) health care system outcomes. The evidence was limited to a literature review by Starfield [68] which found associations with governance, economics, comprehensiveness, population health, and quality. It was shown that investments in primary care produce more equity than investments in the health care system in general. A major source for many types on inequities in health lays in poor maternal health, and infant/child infections. It was also shown that policies targeting average health are not necessarily associated with reduced inequities in health.

## Discussion

### Primary care as a multidimensional system

Primary care is a major research area, as shown by the high number of identified publications. A third of the studies included systematic literature reviews. This provides a sound evidence base for the reported findings. Almost half of the included studies were concerned with only single dimensions of primary care. Though these studies are useful and necessary for increasing our understanding of dimensions, they lack insight into the complexity of primary care. This review was able to provide insight in the complexity of primary care as a multidimensional system, by identifying ten core dimensions that constitute a primary care system, on three levels. The structure of primary care is determined by its governance, economic conditions, and workforce development. The process of primary care is shaped by access to primary care services, the provided scope of services (comprehensiveness), continuity, and coordination of care. A hierarchy of importance could be argued at process level. It is reasonable to assume that the primary care process starts with patients having access to the primary care system. Once a patient has the opportunity to enter the primary care process, it is important that the patient receives appropriate care (quality of care dimension). This is a question of which services are offered to patients. Consequently, the care offered to patients should be delivered in a coordinated manner, on a continuous basis. These two dimensions of coordination and continuity of care are to a great extent interrelated.

This hierarchy of process dimensions can facilitate future measurement studies of primary care process, organization or performance, for example by assigning weights to dimensions.

The outcome of a primary care system is characterized by the provided quality and efficiency of care, and the achieved equity in health. Primary care equity in health received least attention in the literature. This could be because health distribution is the result of many factors, both within and beyond the health care system.

### Evidence for the relevance of primary care dimensions

There is a considerable amount of evidence showing the relevance of the governance and economic conditions of a primary care system. Both dimensions (through primary care supportive governmental policies, universal financial coverage, and low or no patient cost sharing) are associated with the primary care process, in terms of access, continuity, coordination and comprehensiveness of care. They are also of influence for the quality and efficiency of primary care, equity in health, costs of care, and the quality of professional life of primary care providers.

Few studies focussed on the relevance of primary care workforce development. The available evidence showed associations (of gender balance and availability of nurses) with access, continuity, comprehensiveness and efficiency of primary care.

At process level, there was clear evidence that access, comprehensiveness, continuity and coordination of care are all associated with each other. Each dimension at process level is associated with quality of care, efficiency of care, health, and primary care strength. With the exception of comprehensiveness of care, they are also all associated with patient satisfaction and costs of care. Furthermore, access shows associations with equity in health, and quality in professional life of primary care providers. Comprehensiveness of care also seemed to be related to equity in health. The level of health and the distribution of health are not necessarily associated. The evidence for the relevance of equity in health could only be based on one literature review.

The evidence showed that the supply of family physicians and their geographic distribution, consultation length, type of after-hours primary care arrangement, waiting time, and targeted service provision are critical features of access that affect primary care outcomes. The duration of a patient-provider relationship and a provider's medical knowledge of a patient are influential features of continuity of care. Important features of care coordination are having a gatekeeping system (first-contact care), referral rates, task substitution, skill mix, practice size and type of specialist outreach model. For comprehensiveness of care these were the provision of a wide range of services, including particularly preventive care services. Avoidable hospitalizations and the prevalence of ambulatory care sensitive conditions are critical features of quality of care. For efficiency of primary care these were activities (time consumption) of generalists in primary care. It was shown that investments in primary care produce more equity than investments in the health care system in general. A major source for many types on inequities in health lays in poor maternal health, and infant/child infections.

Future research is particularly recommended on primary care workforce development, and possible relations with primary care structure (e.g. governance, financing) and outcome measures. Furthermore, more research is needed on strategies to improve equity in health through primary care.

### Limitations

This review includes only published peer-reviewed studies, and is thus susceptible to publication bias. It excluded hand searching, grey literature and foreign language journals, and was limited to a five year time period due to funding constraints. This may have led to relevant omissions. For reasons of efficiency, this review had a major focus on systematic reviews, assuming they provide an overview of results from other publications. As a result, original research excluded from literature reviews might have been missed. The included original studies had on average an internal validity ranging from fairly strong to weaker, and an average external validity ranging form strong to weaker. We find that the quantitative aspects of studies carried more weight in the total validity score than the qualitative aspects, while descriptive studies form a major part of the primary care research area.

The main difficulty in interpreting the included studies is the lack of proven causalities between primary care dimensions and outcome measures. The evidence is limited to associations and key findings.

## Conclusions

It can be concluded that a primary care system can be defined and approached as: a multidimensional system structured by primary care governance, economic conditions, and a primary care workforce development, facilitating access to a wide range of primary care services in a coordinated way, and on a continuous basis, by applying resources efficiently to provide high quality care, contributing to the distribution of health in the population.

Primary care contributes through its dimensions to overall health system performance and health.

## Competing interests

The authors declare that they have no competing interests.

## Authors' contributions

DK performed the literature review and wrote the manuscript. WB was a reviewer and co-author of the manuscript. AH provided advise on the search strategy and data extraction, and reviewed drafts of the manuscript. PP and JvdZ reviewed drafts of the manuscript. All authors read and approved the final manuscript.

## Pre-publication history

The pre-publication history for this paper can be accessed here:

http://www.biomedcentral.com/1472-6963/10/65/prepub

## Supplementary Material

Additional file 1 **Search strategy**. The strategy used in the MEDLINE search, which was adapted for use in the other databases. Click here for file

Additional file 2 **Characteristics of included studies**. A description of the characteristics of the 85 included studies, including setting, sample size, study description, study focus, and primary care dimension(s) studied. Click here for file

Additional file 3 **Primary care governance**. Key findings for primary care governance and its relation with primary care dimensions and outcomes. Click here for file

Additional file 4 **Economics of the primary care system**. Key findings for economics of the primary care system and its relation with primary care dimensions and outcomes. Click here for file

Additional file 5 **Primary care workforce development**. Key findings for PC workforce development and its relation with PC dimensions and outcomes. Click here for file

Additional file 6 **Access to primary care services**. Key findings for access to primary care services and its relation with primary care dimensions and outcomes. Click here for file

Additional file 7 **Continuity of primary care**. Key findings for continuity of primary care and its relation with primary care dimensions and outcomes. Click here for file

Additional file 8 **Coordination of primary care**. Key findings for coordination of primary care and its relation with primary care dimensions and outcomes. Click here for file

Additional file 9 **Comprehensiveness of primary care services**. Key findings for comprehensiveness of primary care services and its relation with primary care dimensions and outcomes. Click here for file

Additional file 10 **Quality of primary care**. Key findings for quality of primary care services and its relation with primary care dimensions and outcomes. Click here for file

Additional file 11 **Primary care efficiency**. Key findings for primary care efficiency and its relation with primary care dimensions and outcomes. Click here for file

Additional file 12 **Equity in health**. Key findings for equity in health and its relation with primary care dimensions and outcomes. Click here for file
