# Supplementary material for: The breadth of primary care: a systematic literature review of its core dimensions
Source: BMC Health Serv Res. 2010 Mar 13;10:65. doi: 10.1186/1472-6963-10-65 (PMC2848652; doi:10.1186/1472-6963-10-65)
Supplement: Additional file 1 — Search strategy. The strategy used in the MEDLINE search, which was adapted for use in the other databases. [file 1472-6963-10-65-S1.DOC]

### Search strategy

The strategy used in the MEDLINE search is given below. This was adapted for use in the other databases.

Search ((((((((("Review "[Publication Type:noexp] OR "Review Literature as Topic"[Mesh:noexp] AND (("2003"[PDat] : "2008"[PDat]) AND (Humans[Mesh])))) AND (((((("Primary Health Care"[Mesh:noexp] OR "Family Practice"[Mesh]) OR "Physicians, Family"[Mesh]) OR "Ambulatory Care"[Mesh:noexp]) OR "Ambulatory Care Facilities"[Mesh:noexp]) OR "Home Care Services"[Mesh:noexp] AND (("2003"[PDat] : "2008"[PDat]) AND (Humans[Mesh]))))) AND (((((((((((("Health Services Accessibility"[Majr:noexp] OR "Continuity of Patient Care"[Majr]) OR "Delivery of Health Care"[Majr:noexp]) OR "Delivery of Health Care, Integrated"[Majr:noexp]) OR "Comprehensive Health Care"[Majr:noexp]) OR "Professional Role"[Majr]) OR "Patient-Centered Care"[Majr]) OR "Social Environment"[Majr]) OR "Social Conditions"[Majr:noexp]) OR "Family Health"[Majr] AND (("2003"[PDat] : "2008"[PDat]) AND (Humans[Mesh])))) OR ((coordinat* AND (("2003"[PDat] : "2008"[PDat]) AND (Humans[Mesh])))) AND (("2003"[PDat] : "2008"[PDat]) AND (Humans[Mesh])))))) OR ((((("Review "[Publication Type:noexp] OR "Review Literature as Topic"[Mesh:noexp] AND (("2003"[PDat] : "2008"[PDat]) AND (Humans[Mesh])))) AND (((((("Primary Health Care"[Majr:noexp] OR "Family Practice"[Majr]) OR "Physicians, Family"[Majr]) OR "Ambulatory Care"[Majr:noexp]) OR "Ambulatory Care Facilities"[Majr:noexp]) OR "Home Care Services"[Majr:noexp] AND (("2003"[PDat] : "2008"[PDat]) AND (Humans[Mesh]))))) AND (((((((((((("Health Services Accessibility"[Mesh:noexp] OR "Continuity of Patient Care"[Mesh]) OR "Delivery of Health Care"[Mesh:noexp]) OR "Delivery of Health Care, Integrated"[Mesh:noexp]) OR "Comprehensive Health Care"[Mesh:noexp]) OR "Professional Role"[Mesh]) OR "Patient-Centered Care"[Mesh]) OR "Social Environment"[Mesh]) OR "Social Conditions"[Mesh:noexp]) OR "Family Health"[Mesh] AND (("2003"[PDat] : "2008"[PDat]) AND (Humans[Mesh])))) OR ((coordinat* AND (("2003"[PDat] : "2008"[PDat]) AND (Humans[Mesh])))) AND (("2003"[PDat] : "2008"[PDat]) AND (Humans[Mesh])))) AND (("2003"[PDat] : "2008"[PDat]) AND (Humans[Mesh])))) AND (("2003"[PDat] : "2008"[PDat]) AND (Humans[Mesh])))) OR ((((((((((((((("Primary Health Care"[Mesh:noexp] AND (("2003"[PDat] : "2008"[PDat]) AND (Humans[Mesh])))) OR (("Family Practice"[Mesh] AND (("2003"[PDat] : "2008"[PDat]) AND (Humans[Mesh]))))) OR (("Physicians, Family"[Mesh] AND (("2003"[PDat] : "2008"[PDat]) AND (Humans[Mesh]))))) OR (("Ambulatory Care"[Mesh:noexp] AND (("2003"[PDat] : "2008"[PDat]) AND (Humans[Mesh]))))) OR (("Ambulatory Care Facilities"[Mesh:noexp] AND (("2003"[PDat] : "2008"[PDat]) AND (Humans[Mesh]))))) OR (("Home Care Services"[Mesh:noexp] AND (("2003"[PDat] : "2008"[PDat]) AND (Humans[Mesh]))))) OR (("Delivery of Health Care"[Mesh:noexp] AND (("2003"[PDat] : "2008"[PDat]) AND (Humans[Mesh]))))) OR (("National Health Programs"[Mesh:noexp] AND (("2003"[PDat] : "2008"[PDat]) AND (Humans[Mesh]))))) OR (("Health Care Sector"[Mesh] AND (("2003"[PDat] : "2008"[PDat]) AND (Humans[Mesh])))) AND (("2003"[PDat] : "2008"[PDat]) AND (Humans[Mesh])))) AND (((((((("Systems Analysis"[Majr:noexp] OR "Models, Organizational"[Majr]) OR "Outcome and Process Assessment (Health Care)"[Majr:noexp]) OR "Outcome Assessment (Health Care)"[Majr:noexp]) OR "Process Assessment (Health Care)"[Majr]) OR "Benchmarking"[Majr]) OR "Quality Indicators, Health Care"[Majr:noexp]) OR "Social Responsibility"[Majr] AND (("2003"[PDat] : "2008"[PDat]) AND (Humans[Mesh])))))) OR ((((((((((((("Primary Health Care"[Majr:noexp] AND (("2003"[PDat] : "2008"[PDat]) AND (Humans[Mesh])))) OR (("Family Practice"[Majr] AND (("2003"[PDat] : "2008"[PDat]) AND (Humans[Mesh]))))) OR (("Physicians, Family"[Majr] AND (("2003"[PDat] : "2008"[PDat]) AND (Humans[Mesh]))))) OR (("Ambulatory Care"[Majr:noexp] AND (("2003"[PDat] : "2008"[PDat]) AND (Humans[Mesh]))))) OR (("Ambulatory Care Facilities"[Majr:noexp] AND (("2003"[PDat] : "2008"[PDat]) AND (Humans[Mesh]))))) OR (("Home Care Services"[Majr:noexp] AND (("2003"[PDat] : "2008"[PDat]) AND (Humans[Mesh]))))) OR (("Delivery of Health Care"[Majr:noexp] AND (("2003"[PDat] : "2008"[PDat]) AND (Humans[Mesh]))))) OR (("National Health Programs"[Majr:noexp] AND (("2003"[PDat] : "2008"[PDat]) AND (Humans[Mesh]))))) OR (("Health Care Sector"[Majr] AND (("2003"[PDat] : "2008"[PDat]) AND (Humans[Mesh])))) AND (("2003"[PDat] : "2008"[PDat]) AND (Humans[Mesh])))) AND (((((((("Systems Analysis"[Mesh:noexp] OR "Models,

Organizational"[Mesh]) OR "Outcome and Process Assessment (Health Care)"[Mesh:noexp]) OR "Outcome Assessment (Health Care)"[Mesh:noexp]) OR "Process Assessment (Health Care)"[Mesh]) OR "Benchmarking"[Mesh]) OR "Quality Indicators, Health Care"[Mesh:noexp]) OR "Social Responsibility"[Mesh] AND (("2003"[PDat] : "2008"[PDat]) AND (Humans[Mesh])))) AND (("2003"[PDat] : "2008"[PDat]) AND (Humans[Mesh])))) AND (("2003"[PDat] : "2008"[PDat]) AND (Humans[Mesh]))))) NOT ("Clinical Trial "[Publication Type] AND (("2003"[PDat] : "2008"[PDat]) AND (Humans[Mesh]))) AND (("2003"[PDat] : "2008"[PDat]) AND (Humans[Mesh]))) AND (hasabstract[text] AND ("2003"[PDat] : "2008"[PDat]) AND (Humans[Mesh]) AND (English[lang])) AND (hasabstract[text] AND ("2003"[PDat] : "2008"[PDat]) AND (Humans[Mesh]) AND (English[lang]))
