# Supplementary material for: The breadth of primary care: a systematic literature review of its core dimensions
Source: BMC Health Serv Res. 2010 Mar 13;10:65. doi: 10.1186/1472-6963-10-65 (PMC2848652; doi:10.1186/1472-6963-10-65)
Supplement: Additional file 2 — Characteristics of included studies. A description of the characteristics of the 85 included studies, including setting, sample size, study description, study focus, and primary care dimension(s) studied. [file 1472-6963-10-65-S2.DOC]

### Characteristics of included studies

##

| **Author(s)** | **Year** | **Setting** | **Sample size** | **Study design** | **Study focus** | **PC Dimension(s) studied****[[1]](#footnote-2)** |
| --- | --- | --- | --- | --- | --- | --- |
| Aakvik, Holmas [90] | 2006 | PC at municipality level in Norway | 406 municipalities | Retrospective cohort study | The impact of employment status and access on health outcomes. | ECO, WFD |
| Amado, Alexandra, Dyson [51] | 2008 | Not restricted. | n.a. | Review of literature | Methods to compare the performance of PC providers. | GOV, WFD, COM, CON, QUA |
| Ansari, Barbetti, Carson et al. [91] | 2003 | PC and hospital care in rural and urban areas of Victoria, Australia. | All hospitals in 200 Statistical Local Areas in Victoria. | Retrospective cohort study | Analysis of Ambulatory Care Sensitive Conditions (ACSCs) as a measure of health outcomes that might vary with access to PC. | ACC, QUA |
| Ansari [53] | 2007 | Not restricted. | n.a. | Review of literature | The concept and usefulness of ACSCs as indicators for quality and access to PC. | QUA |
| Ansari [52] | 2007 | Not restricted. | n.a. | Review of literature | The meaning and concept of access in the area of PC. | ACC, QUA |
| Arah, Westert, Hurst et al. [54] | 2006 | Not restricted. | n.a. | Review of literature | The development of a conceptual framework for the OECD’s Health Care Quality Indicators project. | ECO, ACC, QUA, EFF |
| Ashworth, Armstrong [17] | 2006 | General practices in the UK. | 8480 general practices | Cross-sectional study | The relationship between quality of care, and social deprivation and GP practice characteristics. | COO, CON |
| Bhat [18] | 2005 | Health care delivery in 24 OECD countries. | None; macro-level data. | Cross-sectional study | The effect of institutional arrangements on efficiency of health care delivery systems. | ECO, COO, EFF |
| Bower, Roland, Campbell et al. [19] | 2003 | General practices in the UK. | 21,905 patients | Cross-sectional study | Patients’ views on access and continuity in general practice. | ACC, CON |
| Bower, Campbell, Bojke et al. [20] | 2003 | Primary care teams in the UK. | 60 PC practices | Cross-sectional study | Relation between team structure, team climate, and the quality of care in PC. | ACC, COO, QUA |
| Branson, Badger, Dobbs [55] | 2003 | Not restricted. | n.a. | Review of literature | Skill mixes in PC that meet patients’ preferences and needs. | WFD, COO |
| Cabana, Jee [56] | 2004 | Not restricted. | n.a. | Review of literature | The effect of sustained continuity of care on the quality of patient care. | CON |
| Campbell, Reeves, Kontopantelis et al. [85] | 2007 | PC practices in the UK. | 60 PC practices | Prospective cohort study | The effect of introducing pay for performance on the quality of PC | GOV, ECO |
| Carmichael, Alvarez, Chaput et al. [92] | 2004 | A PC pharmacy system in a medical center in the Veterans Health Administration in the USA. | 1300 care facilities | Retrospective cohort study | Pharmacist’s contributions to drug therapy within a PC team. | COO |
| Chapman, Zechel, Carter et al. [57] | 2004 | PC in the UK. | n.a. | Review of literature | The evidence of recent innovations in service provision to improve access or equity in access to PC. | ACC |
| Chlabicz, Marcinowicz [21] | 2005 | Two PC centers in Poland. | 1000 patients | Cross-sectional study | Relation between ownership status of PC and quality of care. | GOV, WFD |
| Christakis, Wright, Zimmerman et al. [22] | 2003 | A paediatric care center in the USA. | 759 patients | Cross-sectional study | Relation between continuity of care and well-coordinated care. | CON |
| Crampton [97] | 2005 | PC organizations in New Zealand. | n.a. | Descriptive study | Exploration of different ownership forms, and community participation in PC. | GOV |
| De Maeseneer, De Prins, Gosset et al. [86] | 2003 | Two health insurance providers in Belgium. | 4800 patients | Prospective cohort study | Relation between provider continuity in family medicine and total health care costs. | CON |

| **Author(s)** | **Year** | **Setting** | **Sample size** | **Study design** | **Study focus** | **PC Dimension(s) studied^Error: Reference source not found^** |
| --- | --- | --- | --- | --- | --- | --- |
| Doran, Fullwood, Kontopantelis et al. [15] | 2008 | General practices in the UK. | 7637 general practices | Retrospective cohort study | Effect of financial incentives on inequalities in the delivery of primary clinical care. | GOV |
| Eggli, Halfon, Chikhi et al. [75] | 2006 | Ambulatory health care in Switzerland. | n.a. | Descriptive study | A conceptual framework for an ambulatory health care information system. | ECO, ACC, QUA, EFF |
| Engels,Campbell, Dautzenberg et al. [23] | 2005 | General practice in Belgium, France, Germany, The Netherlands, Switzerland, UK. | 21 GPs, researchers, PC experts | Cross-sectional study | A framework for general practice management made up of quality indicators. | GOV, WFD, ACC, CON, COM, QUA |
| Friedberg, Coltin, Pearson et al. [24] | 2007 | PC physician groups in the USA. | 4358 PC providers | Cross-sectional study | Relation between group size and affiliation with networks of multiple groups with quality of care. | COO, QUA |
| Gené-Badia, Escaramis-Babiano, Sans-Corrales et al. [87] | 2007 | PC teams in Spain. | 257 PC teams (3439 physicians; 3781 nurses) | Prospective cohort study | Impact of economic incentives on quality of professional life and on end-user satisfaction in PC. | GOV |
| Gené-Badia, Ascaso, Escaramis-Babiano et al. [25] | 2007 | PC teams in Spain. | 213 PC teams | Cross-sectional study | Components of PC output that best serve to define the outcome of Family Medicine services. | ACC, COO, QUA |
| Gené-Badia, Ascaso, Escaramis-Babiano, et al. [26] | 2008 | PC teams in Spain. | 213 PC teams | Cross-sectional study | Relation between PC team’s structure and population characteristics with quality of services. | COO, QUA |
| Goodman, Ross, Mackenzie et al. [76] | 2003 | District nursing in the UK, Australia, USA, Far East. | n.a. | Descriptive study | The role and contribution of district nursing to PC. | WFD |
| Green, Ross, Mirzoev [77] | 2007 | PC in the UK. | n.a. | Descriptive study | Assessing the English health system against the Alma Ata PC principles as an evaluative framework. | GOV, EQU |
| Green , Fortin, Maclure et al. [88] | 2006 | A chronic disease management collaborative of PC physicians in Canada. | 30 Community- based physicians | Prospective case study | An information system to improve and support chronic disease management in PC. | CON |
| Grol, Giesen, Van Uden [78] | 2006 | PC cooperatives in the UK, Denmark, the Netherlands. | n.a. | Descriptive study | Models used for organising after-hours care in PC. | ACC |
| Gruen, Weeramanthri, Knight et al. [58] | 2003 | Not restricted. | n.a. | Review of literature | Effectiveness of specialist outreach on health system performance and health outcomes. | COO |
| Grytten, Sorensen [27] | 2007 | General practice in Norway. | 3355 PC physicians | Cross-sectional study | Relation of list size and per capita payment with access to PC and providers’ service production. | CON |
| Haggerty, Burge, Levesque et al. [28] | 2007 | PC in Canada. | 20 PC experts | Cross-sectional study | PC attributes and their operationalisation. | GOV, ACC, CON, COO, COM, QUA, EQU, EFF |
| Halcomb, Davidson, Daly et al. [60] | 2005 | General practice in Australia. | n.a. | Review of literature | The contribution of practice nurses to PC. | GOV, WFD |
| Hanson, Yip, Hsiao [29] | 2004 | Outpatient care in Cyprus. | 8270 individuals | Cross-sectional study | Effect of quality on patients’ choice between public and private outpatient care. | ACC, CON, QUA, EFF |
| Hebrang, Henigsberg, Erdeljic et al. [30] | 2003 | PC in Croatia. | 267 general practices | Cross-sectional study | Effect of privatization on PC accessibility. | ECO |
| Hogg, Rowan, Russell et al.[16] | 2008 | PC organizations in Canada. | n.a. | Descriptive study | A framework to conceptualize the structure, organization and performance of PC. | GOV, ECO, WFD, ACC, QUA |
| Hollinghurst, Horrocks, Anderson et al. [93] | 2006 | PC in the UK. | n.a. | Economic cost analysis | The cost of PC provided by nurse practitioners compared with that of salaried GPs. | COO |
| Hung [31] | 2007 | PC practices in the USA. | 124 PC practices | Cross-sectional study | The contribution of PC practices to the delivery of preventive care. | WFD, COO, CON, COM |
|  |  |  |  |  |  | *(Continued)* |

| **Author(s)** | **Year** | **Setting** | **Sample size** | **Study design** | **Study focus** | **PC Dimension(s) studied^Error: Reference source not found^** |
| --- | --- | --- | --- | --- | --- | --- |
| Hutchison, Ostbye, Barnsley et al. [89] | 2003 | PC and emergency care in Canada. | 12 walk-in clinics, 16 family practices, 13 emergency departments. | Prospective cohort study | Patient satisfaction and quality of care in walk-in clinics, family practices, emergency departments. | ACC |
| Jee, Cabana [60] | 2006 | Not restricted. | n.a. | Review of literature | Indices for continuity of care. | CON |
| Kerssens, Groenewegen, Sixma et al. [32] | 2004 | PC in 9 European countries, and Belarus, Ukraine, Israel. | 5133 patients | Cross-sectional study | Patient evaluations of quality of PC. | COO, QUA |
| Kroneman, Maarse, Van der Zee [33] | 2006 | PC in 18 European countries. | 36 PC experts | Cross-sectional study | Relation between direct access in PC and patient satisfaction. | COO |
| Kuusela, Vainiomaki, Hinkka et al. [34] | 2004 | Health care centres in Finland. | 81 GPs and their patients in 4 health care centres | Cross-sectional study | Comparison of the quality of GP consultations between two Finnish employment contract systems. | ECO |
| Lanier, Roland, Burstin et al. [79] | 2003 | Health care system in the UK, USA and the Netherlands. | n.a. | Descriptive study | Efforts to measure and improve doctors’ performance. | GOV |
| Lee, Kiyu, Milman et al. [80] | 2007 | Not restricted. | n.a. | Descriptive study | Improving the effectiveness of PC. | WFD, ACC, CON, COM, QUA |
| Leibowitz, Day, Dunt et al. [61] | 2003 | Not restricted. | n.a. | Review of literature | The effect of different models of out-of-hours PC service on outcome. | ACC |
| Levaggi, Rochaix [94] | 2007 | Not restricted. | n.a. | Economic cost-benefit analysis | Issues that need to be addressed when access rules are being chosen or reformed, given the type of provider payment. | ECO, COO, EFF |
| Macinko, Starfield, Shi [4] | 2003 | PC in 18 wealthy OECD countries | n.a. | Cross-sectional study | The contribution of PC systems to a variety of health outcomes. | GOV, ECO, WFD, ACC, CON, COO, COM, QUA |
| Marshall, Klazinga, Leatherman, et al. [62] | 2006 | PC systems in OECD countries. | 11researchers/policymakers from 9 OECD countries | Review of literature and consensus procedure | Quality indicators to assess the performance of PC systems. | COM, QUA |
| McDonald, Davies, Cumming et al. [81] | 2007 | PC in the UK, New Zealand and Australia. | n.a. | Descriptive study | The role of Divisions of General Practice and PC Partnerships in addressing Australian challenges. | WFD |
| McInnes, Saltman, Kidd [35] | 2006 | General practice in Australia. | 3000 GPs | Cross-sectional study | The use of computers by GPs for clinical purposes. | GOV, CON |
| Meads, Iwami, Wild [36] | 2005 | PC in 10 countries. | n.a. | Cross-sectional study | Comparing international health systems development in relation to the advent of new PC organizations in countries with parallel ‘modernizing’ policies. | GOV, WFD |
| Naithani, Gulliford,Morgan [37] | 2006 | General practices in the UK. | 25 patients from 14 general practices | Cross-sectional study | Patients’ experiences and values with respect to continuity in diabetes care. | CON |
| Nelson, Simic, Beste et al. [38] | 2003 | PC in Serbia | PC providers /administrators from 13 health care institutions. | Cross-sectional study | A multimodal assessment using health care in post-conflict Serbia as a model. | GOV, ECO, WFD, ACC, EFF |
| Nolte, Bain, McKee [39] | 2006 | Health systems in 29 industrialized countries. | n.a. | Cross-sectional study | Assessment of performance of health systems using diabetes as a tracer condition. | QUA |
| Nutting,Goodwin, Flocke et al. [40] | 2003 | Family practice in the USA. | 4,454 outpatient visits to 138 FPs | Cross-sectional study | Association among patient and visit characteristics and continuity of care. | CON |
| Parchman, Noel, Lee [41] | 2004 | South Texas Veteran’s health care system | 720 patients | Cross-sectional study | The relation between attributes of PC and health care system hassles among veterans with chronic diseases. | COO |
| Parkerton, Smith, Straley [42] | 2004 | Medical clinics of single group model HMO in the USA. | 194 FPs and general internists | Cross-sectional study | The influence of practice coordination and physician continuity on patient outcomes. | COO, CON |
|  |  |  |  |  |  | *(Continued)* |

| **Author(s)** | **Year** | **Setting** | **Sample size** | **Study design** | **Study focus** | **PC Dimension(s) studied^Error: Reference source not found^** |
| --- | --- | --- | --- | --- | --- | --- |
| Peckham [82] | 2006 | PC in the UK. | n.a. | Descriptive study | The recent growth of PC in the UK, and recent developments in health care practice. | GOV, WFD, ACC, COO, QUA, EFF |
| Pink, Brown, Studer et al. [63] | 2006 | Health care systems in the UK, USA, Canada, Australia. | n.a. | Review of literature | The design and considerations of pay-for-performance programs. | GOV |
| Rhydderch, Edwards, Elwyn et al. [64] | 2005 | Not restricted. | n.a. | Review of literature | Organizational assessment in general practice and implications for quality improvement. | GOV |
| Sans-Corrales, Pujol-Ribera, Gene-Badia et al. [65] | 2006 | Not restricted. | n.a. | Review of literature | Relation between the attributes of family medicine and the outcomes of health care provision. | ACC, CON, COO, COM |
| Saultz [66] | 2003 | Not restricted | n.a. | Review of literature | Defining and measuring interpersonal continuity of care. | CON |
| Schellevis, Westert, De Bakker [43] | 2005 | General practice in the Netherlands | 104 general practices, incl. 195GPs | Cross-sectional study | The role of general practice in the Dutch health care system. | GOV, ACC, CON, COO, EFF |
| Scrivens [96] | 2007 | Health care system in the UK | n.a. | Descriptive study | The design of an effective regulatory system. | GOV |
| Shen, Andersen, Brook et al. [44] | 2004 | Family practice in the USA. | 836 FPs | Cross-sectional study | Effects of payment method on clinical decision-making. | ECO |
| Shi, Starfield, Xu et al. [45] | 2003 | PC in community health centres and HMOs in the USA. | 890 patients | Cross-sectional study | PC quality in Community Health Centres and Health Maintenance Organizations. | GOV, ACC, CON, COO, COM |
| Shield, Campbell, Rogers et al. [46] | 2003 | Primary mental health care in the UK. | 115 panellists | Cross-sectional study | Quality indicators for PC mental health services to facilitate quality improvement. | GOV, WFD, ACC, COO |
| Sibthorpe, Gardner [83] | 2007 | PC in Australia. | n.a. | Descriptive study | A conceptual framework to underpin the potential development of a quality system for PC. | GOV, WFD, COM |
| Simoens, Giuffrida [47] | 2004 | Health care system in 18 OECD countries. | Experts in 22 OECD countries | Cross-sectional study | Policies on physician payment methods to promote an efficient deployment of physicians. | ECO, EFF |
| Smith, Allwright, O'Dowd [67] | 2007 | Not restricted. | n.a. | Review of literature | Effectiveness of shared care for chronic conditions. | CON, COO |
| Souliotis, Lionis [84] | 2004 | PC system in Greece. | n.a. | Descriptive study | Creating an integrated health care system in Greece. | GOV, ECO, CON, COO, COM |
| Starfield, Shi, Macinko [13] | 2005 | Not restricted. | n.a. | Review of literature | The contribution of PC to health systems and health. | GOV, ECO, WFD, ACC, CON, COO, COM, QUA |
| Starfield [68] | 2006 | Not restricted. | n.a. | Review of literature | Equity in health and policy recommendations. | GOV, ACC, COM, EQU, QUA, EFF |
| Stille, Jerant, Bell et al. [69] | 2005 | Not restricted. | n.a. | Review of literature | Coordination of care and its effectiveness. | CON, COO |
| Stokes, Tarrant, Mainous et al. [48] | 2005 | General practice in the UK, USA, and the Netherlands. | 1523 GPs/FPs | Cross-sectional study | The value of personal continuity as perceived by GPs and patients in different countries. | WFD, COO, CON |
| Svab, Rotar Pavlic, Radic et al. [49] | 2004 | General practice in 14 Eastern European countries. | n.a. | Cross-sectional study | The status of family medicine in Central and Eastern European countries. | WFD, ACC |
| Van Servellen, Fongwa, Mockus D'Errico [70] | 2006 | Not restricted. | n.a. | Review of literature | Relation of continuity of care with quality care indicators. | CON |
| Van Uden, Ament, Voss et al. [95] | 2006 | Out-of-hours primary care in the Netherlands. | n.a. | Economic cost-benefit analysis | The costs of different models of GP cooperatives. | ACC |
| Verhaak, Brink-Muinen, Bensing et al. [50] | 2004 | General practice in six European countries. | 190 GPs | Cross-sectional study | The effect of different health care systems on demand and supply for psychological help. | COO, COM, QUA |
| Wilhelmsson, Lindberg [71] | 2007 | Not restricted | n.a. | Review of literature | Preventive and health promotive work performed by nurses in PC. | COO, COM |
| Wilson, Childs [72] | 2003 | Not restricted | n.a. | Review of literature | Relation between consultation length, process and outcomes in general practice. | WFD, ACC, QUA, EFF |
| Worrall, Knight [73] | 2006 | Not restricted | n.a. | Review of literature | The importance of interpersonal continuity of PC for elderly people with chronic diseases. | CON |
| Xyrichis, Lowton [74] | 2008 | Not restricted | n.a. | Review of literature | Interprofessional teamworking in primary and community care. | GOV, COO |

1. GOV=Governance of the PC system; ECO=Economic conditions of the PC system; WFD=PC Workforce development; ACC=Access to PC services; COM=Comprehensiveness of PC; CON=Continuity of PC; COO=Coordination of PC; QUA=Quality of PC; EQU=Equity in health; EFF=Efficiency of PC. [↑](#footnote-ref-2)
