# Supplementary material for: The breadth of primary care: a systematic literature review of its core dimensions
Source: BMC Health Serv Res. 2010 Mar 13;10:65. doi: 10.1186/1472-6963-10-65 (PMC2848652; doi:10.1186/1472-6963-10-65)
Supplement: Additional file 3 — Primary care governance. Key findings for primary care governance and its relation with primary care dimensions and outcomes. [file 1472-6963-10-65-S3.DOC]

# Primary care governance

| **Key findings for PC governance and its relation with PC dimensions and outcomes** *(literature review references are in bold)* |
| --- |
| *Access*   - PC supportive governmental policies are positively associated with the adequate access of care **[13]**. - Community-governed non-profit PC practices have lower financial and cultural barriers to access, and a longer duration of visits, compared to for-profit counterparts [97]. |
| *Continuity*   - PC supportive governmental policies are positively associated with the adequate continuity of care **[13]**. |
| *Coordination*   - PC supportive governmental policies are positively associated with the adequate coordination of care **[13]**. - Community-governed non-profit PC practices are more likely to have larger and more diverse staff teams on average, compared to government-owned or for-profit PC practices [97]. |
| *Comprehensiveness*   - Health policies that strengthen PC are associated with increased delivery of wide range of services, and in particular preventive care **[**13,68]. - Inadequate equipment, supplies, and medications are one of the impediments to delivery of PC services [38]. - Community-governed non-profit PC practices are more likely to serve the diverse needs of minority populations compared to government-owned or for-profit PC practices [97]. |
| *Quality*   - Countries with health policies more conducive to PC practice achieve better quality of practice [68]. - Health care legislation is important to protect individuals and communities from harm, and to provide incentives for health care professionals to maintain and/or improve a certain level of service quality [96]. - Quality improvement strategies aimed at the individual doctor produce only modest effects. Professional leadership and centralised regulation of quality improvement are important in creation of a culture that values good professional practice, and might generate professional improvement more quickly than more privatised decentralised approaches [79]. - Pay for performance schemes provide financial incentives that can change professional behaviour and improve the quality of care [63,85]. - Patients receive higher quality care in geographical areas where performance measures and monitoring has been established [85]. - Private family practices provide a higher quality of care than public family practices [21]. |
| *Equity in health*   - Investments in PC produce more equity than investments in the health system in general [68]. - Policies directed at infants and children have much greater long term effect than policies directed at older individuals or populations, because of   the influence of early health on later health [68].   - The effect of PC on improving equity on health depends on the availability of information about the patient needs in the various areas in which PC practices are located **[13]**. - Financial incentive schemes targeting specific diseases have the potential to make a substantial contribution to   the reduction of inequalities in the delivery of clinical care where inequalities are related to area deprivation [15]. |
| *Population health*   - Health policies that strengthen PC are associated with better levels of health [68]. |
| *Local accountability*   - Decentralization of power with the health care decision making system away from central government to local service delivery creates greater local accountability of services to local populations [96]. |
| *Quality of professional life*   - Financial incentives related to annual quality targets may increase physicians’ perception of burden and it may have a negative impact on consumer satisfaction. Incentives on long-term professional development are related to an increase in professionals’ perception of support from the management structure [87]. |
| *Costs*   - Rapidly rising costs may result, at least in part, from performance bonuses for physicians who are not returning a sufficient benefit in terms of outcomes and efficiency [63]. |
| *Strength of PC*   - The most consistent policy characteristics in countries with a strong PC system is the government’s attempts to distribute resources equitably, universal financial coverage, and low or no patient cost sharing for PC services **[13]**. |
