# Supplementary material for: The breadth of primary care: a systematic literature review of its core dimensions
Source: BMC Health Serv Res. 2010 Mar 13;10:65. doi: 10.1186/1472-6963-10-65 (PMC2848652; doi:10.1186/1472-6963-10-65)
Supplement: Additional file 4 — Economics of the primary care system. Key findings for economics of the primary care system and its relation with primary care dimensions and outcomes. [file 1472-6963-10-65-S4.DOC]

**Economics of the primary care system**

| **Key findings for Economics of the PC system and its relation with PC dimensions and outcomes** |
| --- |
| *Access*   - Employed general practitioners (GPs) have weaker incentives to see patients when compared with contracted GPs, resulting in poorer access to care [90]. - Providers adjust the level of offered accessibility benefits according to the intensity of market competitiveness. PC providers with a capitation based contract with a national health insurance institution extend accessibility by structural improvements that are not time consuming such as offering timely appointments, scheduled visits by telephone and providing telephone advice outside working hours [30]. However, time-based (salaried) remunerated GPs provide consultations with a longer duration than capitation-based GPs do [34]. |
| *Continuity*   - Capitation based group patients receive an appointment with the preferred doctor more often than patients in a time-based (salaried) group [34]. |
| *Comprehensiveness*   - Poor financial investment and discouraging worker salaries are one of the impediments to delivery of PC [38]. |
| *Quality*   - Employed GPs provide poorer quality of care compared with contracted GPs [90]. - Capitation based GPs rate their work quality higher than time-based (salaried) remunerated GPs. There are no differences between the groups in the patients’ opinions on the quality of care [34]. - Physicians on average tend to perform less discretionary care under capitated arrangements compared with traditional FFS. Treatment options that offer large, undeniable benefits to the patient are not affected by payment method. Treatments offering relatively small or questionable benefits to the patient are affected by payment method [44]. |
| *Efficiency*   - Institutional arrangements have an impact on the efficiency of the health care system. Countries in which physicians are paid wages and salaries or capitation have higher efficiency than fee-for-service countries [18]. - Fee for service (FFS) payment creates the incentive for physicians to stimulate the provision of medical services, leading to high prices, high rates of unnecessary service use and rising expenditures, but lower rates of referral and volume of prescriptions. Under capitation or salary, physicians have an incentive to maximise their income by under-providing services by selecting or referring of patients (on their health status) or prescription of drugs. Salary payment is associated with fewer tests and referrals than both fee for service and capitation. There are also fewer patient procedures per patient, lower throughput of patients per physician, longer consultations and more preventive care when compared with FFS alone. Flexible blended payment methods based on the combination of a fixed component, through either capitation or salary, and a variable component, through FFS, may produce a desirable mix of incentives [47]. |
| *Population health*   - An increased number of self-employed contract GPs relatively to GPs employed by the municipality on fixed salary contracts, has a positive effect on mortality rates [90]. |
| *Quality of professional life*   - Physicians feel more discomfort or distressed when making clinical decisions under capitation than under FFS payment arrangements [44]. |
