# Supplementary material for: The breadth of primary care: a systematic literature review of its core dimensions
Source: BMC Health Serv Res. 2010 Mar 13;10:65. doi: 10.1186/1472-6963-10-65 (PMC2848652; doi:10.1186/1472-6963-10-65)
Supplement: Additional file 5 — Primary care workforce development. Key findings for PC workforce development and its relation with PC dimensions and outcomes. [file 1472-6963-10-65-S5.DOC]

**Primary care workforce development**

| **Key findings for PC workforce development and its relation with PC dimensions and outcomes** *(literature review references are in bold)* |
| --- |
| *Access*   - Female GPs have longer consultation compared to male GPs **[72]**. |
| *Continuity*   - Female GPs are more likely to engage in ‘active and passive counselling’ and place a higher value on personal continuity compared to male GPs [4**8,**72]. - The wider fragmentation of range of PC providers threatens the continuity of care. A response might be the development of patient held records where patients maintain their own continuity of care [82]. |
| *Comprehensiveness*   - Female GPs offer more lifestyle advice compared to male GPs **[72]**. - An increase in practice nursing availability in general practice has been associated with the enhancement of available services in general practice, such as chronic illness management, wound care and health promotion **[59]**. - Few opportunities for professional development, and little emphasis on or respect for PC are one of the impediments to delivery of primary care [38]. |
| *Efficiency*   - Female GPs investigate more and prescribe less compared to male GPs **[72]**. |
