# Supplementary material for: The breadth of primary care: a systematic literature review of its core dimensions
Source: BMC Health Serv Res. 2010 Mar 13;10:65. doi: 10.1186/1472-6963-10-65 (PMC2848652; doi:10.1186/1472-6963-10-65)
Supplement: Additional file 6 — Access to primary care services. Key findings for access to primary care services and its relation with primary care dimensions and outcomes. [file 1472-6963-10-65-S6.DOC]

**Access to primary care services**

| **Key findings for Access to PC services and its relation with PC dimensions and outcomes** *(literature review references are in bold)* |
| --- |
| *Continuity*   - Faster GPs (average consultation length of less than 7 minutes) record sparser medical histories. Slower GPs arrange follow-up consultations in fewer consultations than faster GPs. Re-consultation rates within four weeks of the index consultation are also lower.**[72]**. - Patient enablement and centeredness are positively correlated with average consultation length **[72]**. |
| *Comprehensiveness*   - A greater supply of family physicians is associated with an earlier detection of breast cancer, colon cancer, cervical cancer, and melanoma. Approaches to preventive care are more generic and result in more improvement in patients’ health status than is the case in specialty-oriented practices. Having a good PC source is the major determinant of receiving even disease-focused preventive care, consisting of blood pressure screening, clinical breast exams, mammograms, and cervical smear tests **[13]**. - GPs with an average consultation length of less than 7 minutes are less likely than slower to recognise and deal with long-term problems, and psychosocial problems, even when controlled for individual consultation length; and engage less in preventive care and health promotion **[72]**. - There is evidence that preventive delivery in specialty practice is inequitable in that it is available preferentially to the more socioeconomically advantaged (at least in Belgium) [68]. |
| *Quality*   - Lack of timely and effective care may have a significant impact on rates of admissions for Ambulatory Care Sensitive Conditions (ACSCs), especially in rural areas, and in lower socioeconomic groups **[53]**. - Geographic areas with more family and general practitioners, have lower hospitalization rates for ACSCs, incl. diabetes mellitus, hypertension, and pneumonia **[13]**. - Deputizing doctors are likely to prescribe less appropriately than doctors from practice-based or co-operative services. GPs prescribe more appropriately than junior emergency medical staff **[61]**. - The number of patients seen per hour is positively associated with prescribing volume, and prescribing quality is positively associated with longer consultation length **[72]**. |
| *Equity in health*   - New contractual arrangements or diversifying modes of provision in PC can enable service provision in formerly under-served areas and   populations. Also, organisation flexibility and targeting services around locally defined needs appears to be effective in improving access for   marginalised groups **[57]**.   - An adequate supply of PC providers reduces disparities in health across racial and socioeconomic groups **[13]**. |
| *Population health*   - Greater supply of PC providers are consistently associated with better health outcomes such as lower rates of all-cause, heart disease, and cancer mortalities, even in the presence of income inequality and other health determinants **[**13,80]. In contrast, a greater supply of specialty physicians is associated with higher mortality **[13]**. - Accessibility of care and consultation time are associated with improvements in the level of population health **[65]**. - The removal of disparities in health care access between higher and local socio-economic groups through targeted public health and health services interventions will have the potential to improve health outcomes in the population and reduce demand on hospital services **[53]**. |
| *Quality of professional life*   - GPs with a high patient-centeredness score have longer average consultations and are more stressed after a higher proportion of these than low scoring doctors. Stress scores are particularly high among slow doctors with high booking rates. No studies examined whether there is a direct association between stress and average consultation length **[72]**. - Models of out-of-hours care have an impact on medical workload. A telephone triage and advice service for after-hours PC may reduce the medical workload. Deputizing services increase immediate medical workload because of the low use of telephone advice and the high home visiting rate. Co-operatives, which use telephone triage and PC centres and have low home visiting rate, reduce immediate medical workload. GPs working in emergency departments may reduce the subsequent medical workload **[61]**. |
| *Patient satisfaction*   - Patients find satisfactory standards of access next day appointments with GPs and a 6-10 minute wait for consultation to begin [19]. - User satisfaction is positively related to consultation time **[65]**. - Studies consistently showed patient dissatisfaction with telephone consultations **[61]**. - Both family practices and walk-in clinics are perceived more positively than emergency departments by patients with regard to perceptions of patient-centred communication, perceptions of the physicians attitude, and delay in the waiting room [89]. |
| *Costs*   - The supply of PC providers is associated with lower total costs of health services, possibly partly because of better preventive care and lower hospitalization rates. In contrast, the supply of specialists is associated with more spending and poorer care **[13]**. - PC practices using telephone triage, advice centres or PC cooperatives to facilitate out-of-hours care are associated with lighter workloads for doctors, fewer face-to-face contacts, and fewer house calls, all connected to fewer costs [78]. - The costs of PC are more dependent on the size of the population the cooperative covers, than on the way the GP cooperative is organised, i.e. separated versus integrated (close to a hospital emergency department) [95]. |
| *Strength of PC*   - The most consistent policy characteristics in countries with a strong PC system are the government’s attempts to distribute resources equitably, universal financial coverage that was either under the aegis of the government or regulated by the government, and low or no patient cost sharing for PC services **[13]**. |
