# Supplementary material for: The breadth of primary care: a systematic literature review of its core dimensions
Source: BMC Health Serv Res. 2010 Mar 13;10:65. doi: 10.1186/1472-6963-10-65 (PMC2848652; doi:10.1186/1472-6963-10-65)
Supplement: Additional file 7 — Continuity of primary care. Key findings for continuity of primary care and its relation with primary care dimensions and outcomes. [file 1472-6963-10-65-S7.DOC]

**Continuity of primary care**

| **Key findings for continuity of PC and its relation with PC dimensions and outcomes** *(literature review references are in bold)* |
| --- |
| *Coordination*   - There a positive association between continuity of care and improved care coordination [2**2,**56]. - A register of appropriate patients increases consultation follow-up and sufficient assessments [71]. |
| *Comprehensiveness*   - Continuity of care improves receipt of preventive services, even after controlling for patients’ age, race, health, and insurance **[13,56,60,**73,80]. |
| *Quality*   - The more clinically complex or vulnerable (chronically ill, extremes of age, less education) the patient is or becomes, the higher the likelihood that a continuity-enriched program is essential to achieve quality care [4**0,**70]. - Continuity of care improves quality of care by decreasing hospitalizations, decreasing emergency department use, improving patient compliance to treatment, fewer errors in diagnosis and treatment, reduced resource consumption, and improving receipt of preventive services **[13,56,60,70,**73,80,86]. - Continuity of care has been consistently documented to improve quality of care for patients with chronic conditions through improved early diagnosis such as asthma and diabetes **[56,**70]. - The length of the patient-provider relationship has been associated with accumulated knowledge of the patient from the physician, and trust in the physician. However, it is not always clear which comes first, continuity of care or quality outcomes **[70]**. - Previous knowledge of a patient, which reflects good continuity of care, increases the doctor’s ability of recognizing psychosocial problems influencing the patient’s health **[13]**. - Very short-term relationships with physicians are associated with poor outcomes, such as high rehospitalisation rates. At least two years of a relationship are generally required for patients and practitioners to get to know each other well enough to provide optimal person-focused care **[13]**. - A freely chosen PC provider provides better assurance of a good relationship than does assigning a practitioner [13]. - The evidence is strong regarding the benefits of an ongoing relationship with a particular provider rather than with a particular place or no place at all **[13]**. - Older people with regular physicians are less likely to be taking many prescribed drugs [80]. |
| *Efficiency*   - Continuity of care has shown to be cost-effective in PC, and ensures greater efficiency of services in time saved in the consultation, less use of laboratory tests, and fewer health care expenditures **[13,65]**. |
| *Population health*   - There a positive association between continuity of care and the level of population health [4,56,65]. Continuity of care consistently shows health benefits such as reduced 5-year mortality rates; reduced deaths associated with hypertension, stroke, and lung cancer; and lower infant mortality [4]. - Patients who lack continuity of care have more sickness [86]. |
| *Patient satisfaction*   - There is a positive association between continuity of care and patient satisfaction **[13,56,60]**. |
| *Costs*   - Continuity of care in children has been demonstrated to reduce later use of health care services, and to reduce health care costs **[73]**. - Provider continuity is associated with a lower total health care costs after controlling for a wide variety of socio-demographic and other patient characteristics, including morbidity [86]. |
| *Strength of PC*   - Continuity of care is a key aspect of a strong PC system **[**13,4]. |
