# Supplementary material for: The breadth of primary care: a systematic literature review of its core dimensions
Source: BMC Health Serv Res. 2010 Mar 13;10:65. doi: 10.1186/1472-6963-10-65 (PMC2848652; doi:10.1186/1472-6963-10-65)
Supplement: Additional file 8 — Coordination of primary care. Key findings for coordination of primary care and its relation with primary care dimensions and outcomes. [file 1472-6963-10-65-S8.DOC]

**Coordination of primary care**

| **Key findings for coordination of PC and its relation with PC dimensions and outcomes** *(literature review references are in bold)* |
| --- |
| *Access*   - Substituting nurses for GPs, or use of telephone for face-to-face consultations may be effective in improving access where GP recruitment and retention is problematic. However, maximising the use of skill mix does not necessarily improve access, as it involves trade-offs between different sorts of access **[57]**. |
| *Continuity*   - The team size must be kept under control, otherwise communication between professionals will become more difficult **[69]**. - Team premises (team locations) are important because they enhance information transaction, facilitate communication, and increase personal familiarity. In contrast, team members having separate bases or buildings can result in them being less integrated with the team, limiting team functioning and effectiveness **[74]**. - Full-time and part-time status is a significant predictor for personal continuity of care, with full-time GPs having more positive attitudes to personal continuity [48]. - Skill mix could be viewed as forming a barrier between doctor and patient, but personal lists and teams where practices are divided into smaller units with shared support may help **[55]**. - A liaison nurse was found to be of value in improving communication between the hospital and general practice. A nurse in this position is also effective in ensuring that patients visit nurse-led follow-up general practices **[71]**. - Shared care improved the recording of risk factors **[67]**. |
| *Comprehensiveness*   - A gatekeeping system with fixed list is favourable for a broad (accessible) pathway to mental health care in PC [50]. - First-contact access and coordinated care are associated with patients’ being up to date on screening, immunization, and health habit-counselling services, after controlling for patients’ age, race, health, and insurance **[13]**. - When referral rates vary widely, both across countries and within them, this suggests either differences in population needs or differences in the comprehensiveness of PC services **[13]**. - Both medical clinic size and shared practice are associated with higher rates of cancer screening and diabetic management examinations [42]. - Nurse practitioners and allied health professionals perform services that address health risk behaviours more often than physicians [31]. |
| *Quality*   - Substitution of the GP by practice nursing in some interdisciplinary tasks helps general practices to reach population-based targets for screening items such as immunisations and health screening **[59]**. - Delegation of screening, assessment and pathology tasks to the practice nurse releases the GP to spend more time with those clients who have greater complex medical needs **[59]**. - PC providers provide at least equal high quality of clinical care as specialists in caring for specific common diseases, and they do better overall when the measures of quality are generic. For less common conditions, the care provided by PC providers with appropriate backup from specialists may be the best. For rare conditions, appropriate specialist care is undoubtedly important, as PC providers would not see such conditions frequently enough to maintain competence in managing them **[13]**. - Specialist interventions are more appropriate (less postoperative complications and episodes) when patients are referred from PC in stead of self-referral **[13]**. - PC team climate (in terms of decision making environment; support for innovation; team discussion; task orientation; clarity of objectives) is positively associated with superior clinical care in diabetes. Team climate is better in single handed practices than in partnerships [20]. - Group practices and teams with a greater occupational diversity are independently associated with a higher quality of care [1**7,**74]. - Physician group affiliation with networks of multiple groups is associated with higher quality. Small groups (practice size) may gain more from network affiliation than larger groups, because they may gain access to quality management expertise, and information technology tools, as well as guideline and decision support tools [24] - Evidence-based practice is often more intensive in teaching teams, in more experienced teams and in those attending population with a lower socio-economic level [26]. - Simple ‘shifted outpatients’ styles of specialist outreach improve access to high quality care, but there is no evidence of impact on health outcomes **[58]**. - Specialist outreach as part of more complex multifaceted interventions involving collaboration with PC (incl. case-conferences, joint consultations, seminars and education sessions) is associated with improved guideline-consistent care (reduced duplication and unnecessary referrals and investigations), and less use of inpatient services, and improved health outcomes **[58]**. - Shared care improves appropriate prescribing and medication adherence and use providing longer term benefits for those at an earlier stage in the disease process **[67]**. |
| *Efficiency*   - Health care systems in which PC physicians act as gatekeeper are found to be more efficient than systems without gatekeepers [18]. - When providers behave as ‘perfect agents’ to patients; no matter the type of provider payment (information symmetry), gatekeeping always   dominates in terms of minimizing financial cost since specialist care is only used when needed. When patients have different time preferences  under information symmetry, efficiency can be enhanced in gatekeeping by giving the patient the option to seek a specialist directly, provided  he/she bears the extra cost.  Under information asymmetry (patient information is imperfect), direct access is shown to be more cost effective. This is due to patients’   ability to constrain the providers’ opportunistic behaviour by ‘voting with their feet’ [94]. |

| **Key findings for coordination of PC and its relation with PC dimensions and outcomes** *(literature review references are in bold)* |
| --- |
| *Efficiency (continued)*   - Larger teams have lower levels of participation compared with smaller sized teams, which is correlated with team effectiveness. Teams with a   high proportion of full-time staff and those who have been working together for longer as a team, are also more effective **[74]**.   - Specialist outreach in PC usually requires additional investment on the part of providers and health care systems when compared with hospital   based care, although the additional costs of outreach may be balanced by improved health outcomes **[58]**.   - Close involvement of generalist clinicians in specialty care leads to more cost-effective care and better health. The generalist should therefore be   involved in the care process as more than just a gatekeeper to specialty care **[69]**. |
| *Population health*   - PC practice coordination is positively associated with patient outcomes [42]. - Specialist outreach as part of more complex multifaceted interventions involving collaboration with PC is associated with improved health outcomes **[58]**. - The coordination of care has mixed results with respect to health outcomes **[65]**. |
| *Patient satisfaction*   - If patients have freedom of choice for the type of HC provider, they evaluate the organizational aspects of GP-services more positively. The existence of a gatekeeping system does not influence patient’s judgements about the quality of the actual care provided by their GP [33]. - PC team climate (in terms of decision making environment; support for innovation; team discussion; task orientation; clarity of objectives) is positively associated with patient evaluations of practice [20]. - Nurse practitioner consultations (task substitution) are associated with improved patient satisfaction [93]. - Many studies in adults and children report that patients and clinicians prefer shared generalist-specialist care **[69]** - PC coordination of care is positively associated with patient satisfaction, particularly for patients with multiple chronic conditions [41]. |
| *Costs*   - Task substitution, by employing a nurse practitioner (NP) in PC is likely to cost much the same (or slightly more) as employing a salaried GP. NPs have a higher number of return consultations compared to GPs. There is considerable variability of qualifications and experience of NPs, which suggests that skill-mix decisions should depend on the full range of roles and responsibilities rather than cost [93]. - Patient direct costs are lower when comparing shared care with hospital outpatient care, mainly due to reduced travel costs **[67]**. |
| *PC strength*   - Coordination of care is positively associated with PC strength [**4,**13]. |
| *(Continued)* |
