# Supplementary material for: The breadth of primary care: a systematic literature review of its core dimensions
Source: BMC Health Serv Res. 2010 Mar 13;10:65. doi: 10.1186/1472-6963-10-65 (PMC2848652; doi:10.1186/1472-6963-10-65)
Supplement: Additional file 9 — Comprehensiveness of primary care services. Key findings for comprehensiveness of primary care services and its relation with primary care dimensions and outcomes. [file 1472-6963-10-65-S9.DOC]

**Comprehensiveness of primary care services**

| **Key findings for comprehensiveness of PC services and its relation with PC dimensions and outcomes** *(literature review references are in bold)* |
| --- |
| *Quality*   - Studies consistently found that lower rates of hospitalization for ambulatory care sensitive conditions are strongly associated with the receipt of a comprehensive scope of PC services **[13]**. |
| *Efficiency*   - The wider the range of services provided by PC providers the better are health outcomes, at lower costs [80]. - Preventive health care activities are cost-effective in the PC setting **[13,65]**. |
| *Equity in health*   - Effective health services directed at early detection and prevention of progression are likely to have a considerable impact in reducing disparities   in severity of illness, whereas interventions outside the health sector are likely to have relatively greater impact on occurrence of illness **[68]**. |
| *Population health*   - PC can influence birth outcomes by essential interventions such as antenatal care, professional care of deliveries, well baby control, immunizations, and common disease treatment [80]. - The provision of a wide range of services provided by PC providers is associated with better objective, as well as self-reported health outcomes **[**13,80]. - Preventive health care activities (particularly when they are not related to any one disease or organ system) are associated with improvements in the level of population health **[13,65,**71]. |
| *Strength of PC*   - Comprehensiveness of PC is positively associated with PC strength in a country [**4,**13]. |
