# Supplementary material for: The breadth of primary care: a systematic literature review of its core dimensions
Source: BMC Health Serv Res. 2010 Mar 13;10:65. doi: 10.1186/1472-6963-10-65 (PMC2848652; doi:10.1186/1472-6963-10-65)
Supplement: Additional file 10 — Quality of primary care. Key findings for quality of primary care services and its relation with primary care dimensions and outcomes. [file 1472-6963-10-65-S10.DOC]

**Quality of primary care**

| **Key findings for quality of PC services and its relation with PC dimensions and outcomes** *(literature review references are in bold)* |
| --- |
| *Governance*   - Preventable hospitalizations and ambulatory care sensitive conditions are an indication of potential inadequacies in PC services, which can be related to mal distribution of PC resources **[52,53]**. |
| *Access*   - Preventable hospitalizations and ACSCs are an indication for potential inadequacies in PC services, which can be related to the existence of barriers to accessing PC services **[52,53]**. |
| *Continuity*   - Preventable hospitalizations and ACSCs are an indication for potential inadequacies in PC services, which can be related to problems in continuity of care **[52]**. |
| *Coordination*   - PC teams that rely on good technical quality (such as the % of hypertensive drugs prescribed) do not necessarily have good outputs in terms of inter-personal relationships with patients, or with respect to team coordination [25]. |
| *Efficiency*   - Although there is a view that some prescribing in general practice is unnecessary, crude rates are difficult to link to quality without evidence of appropriateness. Similarly, investigation, referral, and re-consultation rates may conceal differences that really matter, such as appropriateness of these actions **[72]**. - Preventable hospitalizations and ACSCs are an indication for potential inadequacies in PC services, which can be related to inefficient use of resources **[52]**. |
| *Population health*   - The conditions bronchitis, asthma emphysema ischemic heart disease pneumonia influenza are particularly sensitive to PC since their population prevalence and severity depend on prevention, early diagnosis, continuous care, and coordination among different levels of care [4]. - Countries with a low quality of PC have poorer health outcomes, most notably for indicators in early childhood, particularly low birth weight and postneonatal mortality, indicating the focus of PC on prevention and early identification of disease [**4,**13]. |
| *Strength of PC*   - Studies consistently show a relationship between PC strength and quality of care (in terms of health outcomes studied), regardless of the year, level of analysis, or type of outcome measured [**4,**13]. |
