# Supplementary material for: The breadth of primary care: a systematic literature review of its core dimensions
Source: BMC Health Serv Res. 2010 Mar 13;10:65. doi: 10.1186/1472-6963-10-65 (PMC2848652; doi:10.1186/1472-6963-10-65)
Supplement: Additional file 11 — Primary care efficiency. Key findings for primary care efficiency and its relation with primary care dimensions and outcomes. [file 1472-6963-10-65-S11.DOC]

**Primary care efficiency**

| **Key findings for PC efficiency and its relation with PC dimensions and outcomes** *(literature review references are in bold)* |
| --- |
| *Economics of the PC system*   - Countries in which physicians are paid wages and salaries or capitation have higher efficiency than fee-for-service countries [18]. - Fee for service (FFS) payment creates the incentive for physicians to stimulate the provision of medical services, leading to high prices, high rates of unnecessary service use and rising expenditures, but lower rates of referral and volume of prescriptions. Under capitation or salary, physicians have an incentive to maximise their income by under-providing services by selecting or referring of patients (on their health status) or prescription of drugs. Salary payment is associated with fewer tests and referrals than both fee for service and capitation. There are also fewer patient procedures per patient, lower throughput of patients per physician, longer consultations and more preventive care when compared with FFS alone. Flexible blended payment methods based on the combination of a fixed component, through either capitation or salary, and a variable component, through FFS, may produce a desirable mix of incentives [47]. |
| *Workforce development*   - Female GPs investigate more and prescribe less compared to male GPs **[72]**. |
| *Access*   - Efficiency in general practice can be achieved by a decrease in the number of home visits, and by a higher number of telephone contacts. However, when there is still a high variation between practices in this respect, this indicates that there is still room for more efficient practice organization [43]. |
| *Continuity*   - Continuity of care has shown to be cost-effective in PC, and ensures greater efficiency of services in time saved in the consultation, less use of laboratory tests, and fewer health care expenditures **[13,65]**. |
| *Coordination*   - Health care systems in which PC physicians act as gatekeeper are found to be more efficient than systems without gatekeepers [18]. - When providers behave as perfect agents to patients; no matter the type of provider payment (information symmetry), gatekeeping always   dominates in terms of minimizing financial cost since specialist care is only used when needed. Under information asymmetry (patient  information is imperfect), direct access is shown to be more cost effective [94].   - Larger teams have lower levels of participation compared with smaller sized teams, which is correlated with team effectiveness. Teams with a   high proportion of full-time staff and those who have been working together for longer as a team, are also more effective **[74]**.   - Specialist outreach in PC usually requires additional investment on the part of providers and health care systems when compared with hospital   based care, although, the additional costs of outreach may be balanced by improved health outcomes **[58]**.   - Close involvement of generalist clinicians in specialty care leads to more cost-effective care and better health. The generalist should therefore be   involved in the care process as more than just a gatekeeper to specialty care **[69]**.   - Efficiency in general practice can be achieved by delegating more tasks to the practice support staff. However, when there is still a high variation between practices in this respect, this indicates that there is still room for more efficient practice organization [43]. |
| *Comprehensiveness*   - The extent to which a wide range of services are provided by PC providers is associated with better health outcomes at lower costs [80]. - Preventive health care activities are cost-effective in the PC setting **[13,65]**. - When referral rates vary widely, both across countries and within them, this suggests either differences in population needs or differences in the comprehensiveness of PC services [43]. |
| *Quality*   - Although there is a view that some prescribing in general practice is unnecessary, crude rates are difficult to link to quality without evidence of   appropriateness. Similarly, investigation, referral, and re-consultation rates may conceal differences that really matter, such as appropriateness  of these actions **[72]**.   - Preventable hospitalizations and ACSCs are an indication for potential inadequacies in PC services, which can be related to inefficient use of resources **[52]**. |
