# Supplementary material for: The breadth of primary care: a systematic literature review of its core dimensions
Source: BMC Health Serv Res. 2010 Mar 13;10:65. doi: 10.1186/1472-6963-10-65 (PMC2848652; doi:10.1186/1472-6963-10-65)
Supplement: Additional file 12 — Equity in health. Key findings for equity in health and its relation with primary care dimensions and outcomes. [file 1472-6963-10-65-S12.DOC]

**Equity in health**

| **Key findings for equity in health and its relation with PC dimensions and outcomes** *(literature review references are in bold)* |
| --- |
| *Governance*   - Policy decisions to improve average levels of health will not necessarily improve equity in health and may even decrease it. Moreover, policy to increase equity in health and resulting in both relative and absolute changes may have little practical impact because of the frequency of the problem addressed is rare in the population **[68]**. |
| *Economics*   - Investments in PC produce more equity than investments in the health system in general **[68]**. - The influence of income inequality on health in particular areas is widely debated. Consensus is emerging that it is place specific and more salient as an influence on health in the US than in most other industrialized countries **[68]**. |
| *Comprehensiveness*   - The basis for many types on inequities in health lies in early life. The two main areas of influence involve poorer maternal health prior to pregnancy and infant/child infections, both of which are more common in socially disadvantaged populations. These early childhood manifestations of poor health have correlates in health at older ages. Social disadvantage is damaging at any stage in life but is especially harmful when experienced early in life. Thus, other things being equal, priority should be given to effective interventions at younger ages **[68]**. |
| *Population health*   - Improving average health is not necessarily associated with better distribution of health (equity). On the whole, the areas in which inequities in health primarily exist are in common manifestations of ill health and in the severity and progression of common illnesses **[68]**. |
| *Quality*   - There is a clear role for appropriate health services in reducing inequities in health, for example, by attacking severity of illness and preventing co-morbidity **[68]**. |
